# Effect of Lipase and Phospholipase A1 on Foaming and Batter Properties of Yolk Contaminated Egg White

**DOI:** 10.3390/foods12061289

**Published:** 2023-03-17

**Authors:** Xiao-Yan Liu, Wei Chen, Cheng-Tao Wang

**Affiliations:** Beijing Advanced Innovation Center for Food Nutrition and Human Health, Beijing Engineering and Technology Research Center of Food Additives, Beijing Technology & Business University (BTBU), Beijing 100048, China; 2030202063@st.btbu.edu.cn (X.-Y.L.); weichen@btbu.edu.cn (W.C.)

**Keywords:** egg white, foaming properties, yolk-contaminated, lipase enzymolysis

## Abstract

Egg white (EW) is frequently used in bakery products because of its excellent foaming capabilities. However, egg yolk (EY) contamination often degrades the foaming characteristics of EW. The purpose of this study was to investigate the effect of different concentrations of phospholipase A1 (PLPA1) and lipase (LP) on EW. The changes in particle size distribution and potential before and after enzymatic digestion of EW with contaminated 0.5 wt% and 1.0%wt EY were tested. The foaming rate and foam stability were measured after the dispersions were digested with different concentrations of PLPA1 and LP. Additionally, the dispersion samples were used to prepare batter and angel cake, and the modulus, density, and microstructure of the batter were analyzed. Results showed that the potential absolute value increased when the EY was hydrolyzed by PLPA1. The distribution of yolk particle size showed a new aggregation and the average particle size decreased after LP hydrolysis. The dispersion samples hydrolyzed by PLPA1 and LP recovered all the properties of the samples at enzymatic concentrations of 500 U/g and 2500 U/g. This may be attributed to the changes in yolk particles resulting from the enzymatic digestion of EY and the production of amphiphilic lysophospholipids, fatty acids, and glycerol.

## 1. Introduction

Liquid foam is defined as two-phase systems, which are composed of a discontinuous gas phase dispersed in a continuous liquid [1]. Two crucial factors affect the foam quality: foam capacity and foam stability. Foam capacity is dependent on the incorporation level of air in the protein solution, which is determined by measuring the increase in foam volume. The final foaming capacity rests on the rate of the protein adsorption and unfolding at the air-water interface [2]. At the same time, the stability of foam is determined by measuring the decline rate of the liquid discharged from foam with time [3]. Both the foam stability and quality are closely related to the interfacial properties of the foam. Properties of the interfacial layers are governed by their composition and structure, but also to a large extent by interfacial intermolecular interactions such as repulsive electrostatic as well as attractive hydrophobic interactions [4]. Egg white (EW) protein has suitable amphiphilic property, which is conducive to its air expansion to form foam and maintain a stable structure. Thus, EW is extensively applied as an excellent foaming agent in aerated foods such as cakes, cookies, mousses, and dessert shells [5].

The mixing of EY can significantly degrade the foaming characteristics of EW. According to Wang’s research [6], even 0.5 wt% EY will destroy the foaming characteristics of egg protein system. Thus, it seems impossible to avoid any EY contamination of EW even with advanced egg cracking and breaking technology. EY contains water (about 50%), lipids (about 34%), and proteins (about 16%). The lipids are mainly triacylglycerols (66%), phospholipids (28%), and cholesterol (5%). The major egg yolk proteins (EYPs) are livetin (30%), phosvitin (11%), and lipoproteins, which are low-density lipoproteins (LDLs) (23%) and high-density lipoprotein (HDLs) (35%) [7,8]. Phospholipases could hydrolyze phospholipids in EY components, and the foaming and structural features of yolk fractions (including the yolk itself, yolk pulp, and yolk pellets) and their hydrolysates (hydrolyzed by lipase and phospholipase A2) were examined. The results of the study indicated that both lipase and phospholipase A2 could enhance the foaming properties of the yolk fraction [9]. However, the two types of the enzyme showed different mechanisms of the effects on foaming properties of yolk fractions. Specifically, compared with lipase, greater foamability and foam stability were achieved when hydrolyzed by phospholipase A2.

Angel food cake is a simple food system traditionally made of EW, sugar and flour. The lack of egg yolk (EY) and oil makes this dish low-fat and low-calorie, and a customer favorite. EW is a main ingredient [10]. The cake preparation could be utilized to determine the foaming capability of albumen [11]. It is of particular interest among food foam products because the protein foam is generated independently and then combined with other ingredients, allowing separate investigation of the properties of the foam and food product. Essentially, it serves as a model system for investigating foam formation and thermal transformation from fluid to solid foam [12]. Therefore, the foaming characteristics of protein could reflect its final characteristics. The ones made with EW foam have a higher volume than similar ones made with whey protein foam [11,12]. Therefore, EW is also the main and ideal raw material for making cakes. According to the effect of the deterioration of EY on the foaming properties of EW, Xin’s study showed the mixing characteristics of 0–1% EY with its batter [13]. When EW was contaminated with EY, even the cake ratio was lower; the end product was denser, bubbles were larger and uneven, and the crumbs in the cake were more compact.

Phospholipase A1 and phospholipase A2 have similar functions but differ in the location of fatty acid hydrolysis. This study determined the effects of different concentrations of phospholipase A1 and lipase on EY and the foaming properties, batter properties, and cake properties of EW samples contaminated with 0.5% and 1% EY, and the mechanisms were hypothesized and analyzed for the purpose of eliminating EY contamination on the foaming and batter properties of industrial EW.

## 2. Materials and Methods

### 2.1. Materials

Fresh hen eggs were purchased from the local supermarket (Deqingyuan, Beijing, China), The egg weighs 50–55 g (weighed with an electronic scale). BCA Protein Assay Kit was purchased from yuanye Bio-Technology Co., Ltd. (Yuanye, Shanghai, China). Low-gluten flour (Zhanyi, Jiaxing, China) and soft white sugar (Zhanyi, Jiaxing, China) were purchased from a local supermarket, Lipase (LP, 100 kU/g) was purchased from Macklin reagent Shanghai Co., Ltd., and phospholipase A1 (PLPA1, 100 kU/mL) was purchased from Aladdin reagent Shanghai Co. Ltd. (secreted by *Aspergillus oryzae*). Water purified by treatment with a HYP-QX Ultra-pure water machine (Huiyipu, Beijing, China), resistivity stabilized at 18.25 MΩ at room temperature. The latter was used as the solvent throughout the experiments, adding 0.02 wt% sodium azide as a bactericide. All other analytical grade chemicals were purchased from Sinopharm Chemical Reagent Co. Ltd. (Shanghai, China).

### 2.2. Sample Preparation

#### 2.2.1. Preparation of Compound EW Solutions and Enzymolysis of EY

Fresh hen eggs were broken manually, and the EW was obtained (pH 7.92, weighed with a pH meter). Protein was carefully separated from EY. The lacing of the separated EW was carefully removed with tweezers, and the yolk membrane was punctured to separate the yolk (pH 6.21, weighed with a pH meter). The separated EW and EY were pre-stirred for 1 h at room temperature to homogenize.

LP was dissolved in 10 mM phosphate buffer (PBS) at pH 8.0 in advance. The method recorded in Yang’s study determined the actual enzyme activity of PLPA1 and LP [14] and contained the following steps: Prepare PLPA1 and LP with PBS (pH 8.0) into a solution of 20,000 U/mL. Add the LP and PLPA1 solutions to the EY solution to keep the enzyme concentration in the EY solution at 500 U/g, 2500 U/g, and 10,000 U/g and hydrolyzed at 40 °C for 1 h. Then quickly put the solution into ice water at 4 °C. Table 1 shows the amount of the various ingredients added in the experiment on enzymatic EY.

The separated EY was added to the separated EW and stirred for half an hour at room temperature (about 25 °C); then various masses of LP and PLPA1 were added to different concentrations of the solution and stirred for 1 h at 40 °C at room temperature (about 25 °C) after enzymatic digestion. After enzymatic digestion, the enzymes were quickly put into a 72 °C water bath for 1 min to inactivate the enzymes and simulate the synchronization of the pasteurization process in the industry. Then the mixture was quickly put into ice water at 4 °C. Table 2 shows the amount of each component added to the enzymatic EY-contaminated EW samples.

#### 2.2.2. Preparation of Batters

Batters were prepared following the modified angel cake recipe described by Guadarrama-Lezama., et al. [15]. Ingredients were: 40 g low-gluten flour, 80 g sugar, and 120 g yolk-contaminated EW samples. In the first step, 120 g egg solutions were whipped for 2 min under mode 5 in a stainless steel LZ508 eggbeater (Zhigao, Guangzhou, China), to obtain wet foams. Secondly, 40 g sugar were added while whipping continued under the same conditions for another 2 min until soft peaks were formed. Finally, the remaining sugar and low-gluten flour were poured into the eggbeater. After adding flour, the batter was mixed in mode 4 (4000 r/min) for 1 min.

#### 2.2.3. Preparation of Cakes

Cakes were prepared according to Chang’s method with slight modifications [16]. Prepared batter (180 g) was transferred into cube baking containers (Side length 102 mm) and baked for 30 min in the oven (CRTF30W, Weishida Electrical Technology Co., Ltd., Foshan, Guangdong, China) at 170 °C and 180 °C, top and bottom temperatures.

### 2.3. Determination of Enzymatic EY

#### 2.3.1. Measurement of Zeta Potential

Zeta-potentials of sample dispersions were measured in the standard folded capillary electrophoresis cells using a Zetasizer Nano ZS instrument (Malvern Instruments, Worcestershire, UK). Before measurement, EY solution and EY enzymolysis solution were diluted with deionized water at 1:20 (*v/v*) and placed in a 4 °C refrigerator for 2 h. Then, secondary dilution was performed according to 1:20 (*v/v*) Each individual zeta-potential data point was reported as the average and standard deviation of triplicate samples.

#### 2.3.2. Particle Size Distribution

The method referring to Li is slightly modified [17]. The particle size distribution (PSD) under different treatments was measured by dynamic light scattering via a Zetasizer Nano-ZS (Malvern Instruments, Worcestershire, UK) at 25 °C. The refractive index of proteins and the aqueous phase was set at 1.45 and 1.33, respectively. Each sample was measured in triplicate. Before measurement, EY and EY enzymolysis solutions were diluted with deionized water at 1:20 (*v/v*) and placed in a 4 °C refrigerator for 2 h. Then a secondary dilution was conducted according to 1:20 (*v/v*). 

### 2.4. Foamability and Foam Stability

Sample solution of 100 g (as shown in Table 2) was whipped with a stainless steel LZ508 eggbeater (Zhigao, Guangzhou, China) under mode 5 for 3 min at room temperature. The mass of 100 mL of foam was measured by gently filling the 100 mL measuring cup with foam and carefully smoothing the foam with a scraper. 

Foam was prepared with the above method, and the final liquid mass was drained from the foam after 30 min of standing at 25 °C; the foam was measured to calculate foaming properties. Equations (1) and (2) calculated the overran and drainage rate.

FC (mL/g) = 100/m_1_ (1)

Drainage rate (%) = m_2_/100 (2)

where FC (mL/g), m_1_ (g) and m_2_ represent foam capacity, the mass of 100 mL foam and drained liquid mass.

### 2.5. Properties of Batter

#### 2.5.1. Rheological Properties of Batter

The method referring to Rafe A was slightly modified [18]. Rheological properties of batters were determined by the MCR102 dynamic rheometer (Anton Paar Instruments, Graz, Stelmark, Austria). The dynamical oscillatory frequency sweep was performed where the frequency varied from 0.1 to 25 Hz. All measurements were conducted at the strain value of 0.5% and 25 °C (within the linear viscoelastic region determined in preliminary strain sweep test). During temperature sweep testing, sample solutions were heated from 25 °C to 95 °C at a rate of 5 °C/min. The strain and frequency were set to 0.1% and 1 Hz, respectively (within the viscoelastic region). Silicone oil was employed to protect against dehydration during the entire experiment.

#### 2.5.2. Microstructure Observation

The microstructure of fresh batter samples (after foaming) were observed by using a Microscope (4 × 10, Olympus Corporation, Tokyo, Japan). One drop of sample was placed on a microscope slide and then covered slightly with a coverslip [19]. The software associated with the digital camera (Olympus Corporation, Tokyo, Japan) was characterized to record the bubble images. Then, Image Analysis System 11.0 (Changheng Rongchuang Technology Co., Ltd., Beijing, China) was used to measure the foam diameter of foam in the captured image.

#### 2.5.3. Specific Density of Batters

The specific density of each batch of cake batter was measured by carefully putting the batter into a measuring cylinder and gently smoothing, measuring the volume of the batter, and using an electronic scale to measure the paste mass below the volume, then calculating the density of the batter by volume and mass.

### 2.6. Properties of Cakes

#### 2.6.1. Specific Volume of Cakes

Cake volume was measured by using a seed displacement method slightly modified, millets instead of rapeseeds [20]. Millets were put in the empty loaf pan to measure the total volume. After baking the angel food cake in the loaf pan, millets were put in to fill the empty volume of the loaf pan. The volume occupied by the angel food cake was obtained by measuring the total and unoccupied volumes. The final height for each formulation was also determined. Angel food cakes were measured from the base to their highest point per the procedure.

#### 2.6.2. Texture Profile Analysis (TPA)

The method referred to by Reza S M was slightly modified [21]. Sample cakes were cut into uniform square shapes (20 × 20 mm). Crumb texture was evaluated by a texture analyzer (TA.XT Plus, Stable Micro systems Ltd., Godalming, UK), which was attached to the computer software Stable Micro Systems (version 6.1.14.0, Godalming, Surrey, UK). In the TPA process, parameters were set as follows: the pretest speed, test speed, and post speed were all set to 1.0 mm/s, and the type of probe was P/36R, and the distance was 50% of original cake height, measured at ambient temperature. Two continuous compressions were performed for each sample and TPA texture attributes (hardness, adhesiveness, cohesiveness, springiness, and chewiness) were extracted from the force–time curve.

### 2.7. Statistical Analysis

Each measurement was conducted in triplicate. Significance among samples was determined using one-way ANOVA with Duncan’s Multiple Range Test by SPSS version 19.0 software (SPSS Inc., Chicago, IL, USA) with *p*-value being set as 0.05. Data were shown as mean ± standard deviation. Correlations among different indicators were carried out using Pearson’s correlation coefficient in bivariate linear correlations using SPSS version 19.0 software (SPSS Inc., Chicago, IL, USA).

## 3. Results

### 3.1. Zeta Potential

**Figure 1:**
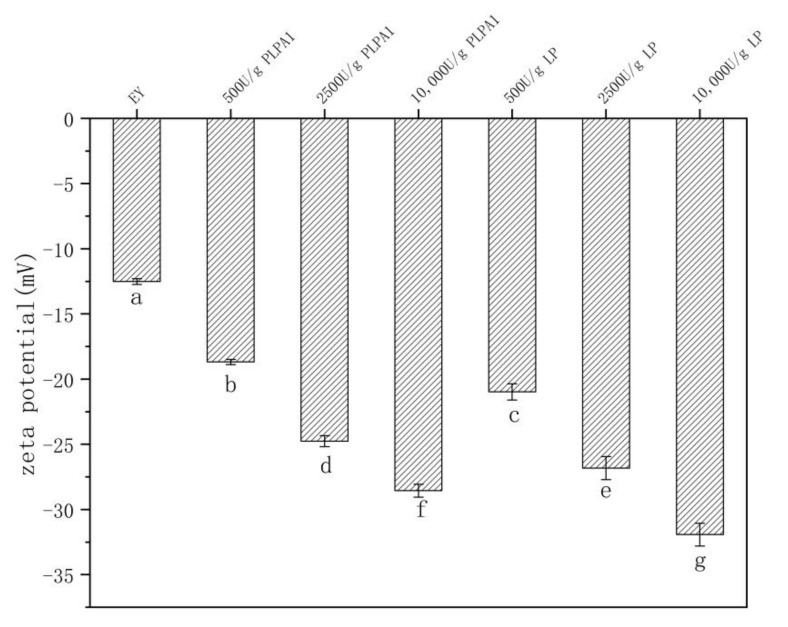
Zeta potential of EY under different concentrations of PLPA1 and LP hydrolysis. (Samples correspond to Table 1). Different letters for the same parameter indicate significant differences (*p* < 0.05) between treatments.

As shown in Figure 1, the potential changes of EY hydrolyzed by PLPA1 and LP showed the same trend as that in the previous study [15]. The zeta potential (absolute value) increased after the hydrolysis of LP and PLPA1. The absolute potential of both PLPA1 and LP increased with enzyme concentration. PLPA1 and LP digestion increased in charged groups exposed on the surface of EY components, with a higher degree of enzymatic digestion indicating a greater degree of charged dot groups exposed on the surface of EY particles [22].

### 3.2. Particle Size 

**Figure 2:**
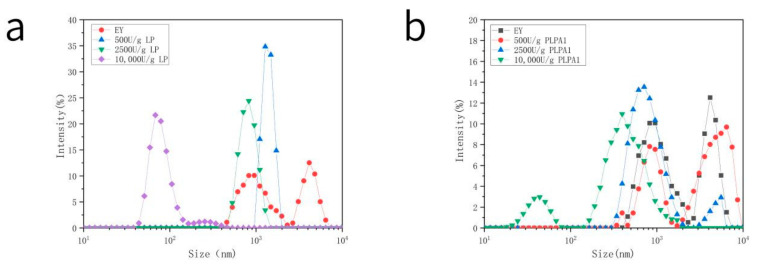
Particle size distribution of EY samples. Samples hydrolyzed by LP (**a**) and PLPA1. (**b**) under different enzymatic hydrolysis concentrations (500 U/g, 2500 U/g, 10,000 U/g).

The results of particle size distribution are shown in Figure 2a. EY has two particle size distributions after enzymatic hydrolysis by lipase, which converged into a single peak particle size distribution, and the particle size decreased with the increase in enzyme concentration. The increase in LP concentration was positively related to the degree of enzymatic hydrolysis increases but negatively related to particle size. Figure 2b compares the effects of PLPA1 at different concentrations on the particle size distribution of EY. It can be seen that after the hydrolysis of PLPA1 at low concentrations, the particle size distribution trend was similar to that of natural EY, but the distribution of the two peaks was wider and slightly increased on average. This may be explained by the fact that a low concentration of PLPA1 was less damaging to the structure of EY, leading to a loose structure of LDL and HDL. An increase in PLPA1 concentration was positively correlated to gradually elevated damage in the structure, and the peaks distributed at 1000–10,000 nm decreased and finally disappeared. When the enzyme concentration was 10,000 U/g, the particle size was mainly 100–1000 nm, and a small peak appeared in the 10–100 nm range. 

PLPA1 and LP hydrolysis caused different decomposition and aggregation of particle groups in EY. Lipase hydrolysis re-aggregated the two original particle sizes (HDL and LDL) in EY into particles in a new particle size. The particle size of such particles decreased with the increase in the degree of enzymatic hydrolysis. Enzymatic hydrolysis of PLPA1 gradually decomposed HDL and LDL. HDL was gradually decomposed by a higher degree of enzymatic hydrolysis as there was a large particle size distribution in the original LDL particle size distribution. LDL could retain the original structural characteristics to a large extent. Low-density lipoprotein was a component of high-density lipoprotein, which could include many LDLs released during HDL decomposition. A higher degree of enzymatic hydrolysis disrupted LDL structure [23].

### 3.3. Foaming Properties

**Figure 3:**
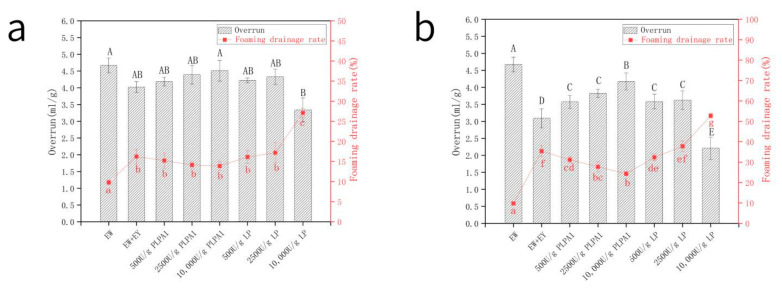
Overrun and foaming drainage rate of samples. Samples mixed with 0.5 wt% EY (**a**) and samples mixed with 1 wt% EY (**b**) hydrolyzed by various concentrations (500 U/g, 2500 U/g, 10,000 U/g) of LP, and PLPA1. (Samples correspond to Table 2). Different letters for the same parameter indicate significant differences (*p* < 0.05) between treatments in the two figures respectively.

It can be seen that the foaming property and foam stability of EW contaminated by 0.5 wt% EY (Figure 3a) and by 1.0 wt% EY (Figure 3b) were significantly reduced, which was the same as the research trend of Li [9]. PLPA1 hydrolysis optimized the foaming and foam stability of yolk-contaminated EW and increased with higher enzyme concentration. As liquid foams are intrinsically unstable, their gas cells should be stabilized to avoid bubble coalescence, disproportionation, and rise [24]. This is typically achieved using surface-active components, which could decrease the interfacial tension between both phases. Surface-active components can be divided into two classes—surface-active lipids and polymers (for example, proteins). Surface-active components are amphiphilic [25]. The LDL represents this fraction’s most flexible surface-active lipids and is readily adsorbed at the interface [26,27]. 

The particle size distribution of EY hydrolyzed by PLPA1 was analyzed. After the hydrolysis of EY by PLPA1, HDL decomposition may release its component LDL and photoprotein. At the same time, PLPA1 cut the ester bond at the SN-1 position of the triglyceride (TG) and converted the phospholipid into lysophospholipids [28]. Lysophospholipid showed good amphiphilic properties and can be better adsorbed at the gas-water interface. This could be explained by the fact that PLPA1 treatment improved foaming property and foam stability. Surface-active lipids were more mobile than surface-active proteins, and they were rapidly moving to the air-water interface, resulting in a fluid-like film and stabilizing gas cells [29]. When both surface-active proteins and lipids are present, they compete at the air–water interface, resulting in a lower foam stability than that containing only protein [30]. The sample foaming property and foam stability cannot be restored to the blank group. LP hydrolysis at a concentration of 500 U/g or 2500 U/g improved the foaming ability and foam stability, while at a concentration of 10,000 U/g, the foaming ability and stability of foam decreased significantly. LP can hydrolyze fatty acids at multiple locations to produce a complex lipid mixture of diacylglycerol (DAG), monoacylglycerol (MAG), glycerin, and fatty acids. Except for MAG, total lipids and all lipids in fat and enzymatic hydrolysates were negatively correlated with foam stability in terms of foaming [31]. Therefore, the production of MAG may be responsible for improving the foaming property and foam stability by lipase hydrolysis at a lower concentration. The formation of fatty acids and glycerol may explain the deterioration of foam characteristics caused by the enzymatic hydrolysis of 10,000 U/g concentration.

### 3.4. Characteristics of Batters

#### 3.4.1. Rheological Properties

Rheological analysis was carried out to provide some information about the characteristics of different batters. The quality of the batter depends on the elastic modulus, the elasticity of foam film is essential in the later stages of baking. When the foam expands with heat, the high elasticity of foam film will prevent it from breaking prematurely during the baking process [27].

**Figure 4:**
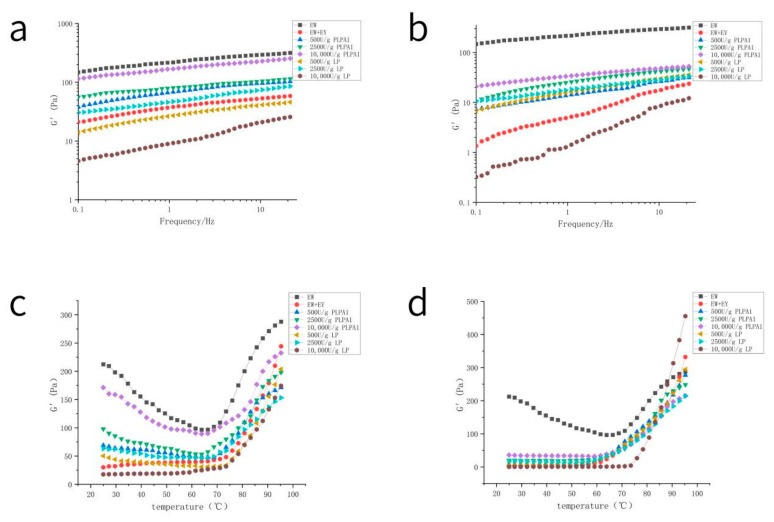
Rheological properties of fresh cake stick. The frequency scanning of fresh cake stick made by PLPA1 or LP hydrolysis of EW contaminated by 0.5 wt% EY (**a**) and 1 wt% EY (**b**); Storage modulus G′development from 25 °C to 95 °C of EW contaminated by 0.5 wt% EY (**c**) and 1 wt% EY (**d**).

It can be seen from Figure 4a,b that the storage modulus was significantly reduced after receiving yolk pollution and that the reduction was more obvious when the concentration of high yolk was 1.0 wt% than 0.5 wt%. This was similar to the research results of Li [17]. Except for the LP hydrolysis of 10,000 U/g, other enzymatic hydrolysis treatments increased the elastic modulus of the sample, and the recovery effect of elastic modulus was better with the increase of enzyme concentration. The effect of PLPA1 was more significant than that of LP. Although it is different from the actual process of baking cake, the change of the elastic modulus of the paste during heating reflected the formation of the network in the paste during heating. 

Furthermore, that change reflects the cake-baking process to some extent. Figure 4c,d show that the initial elastic modulus of the sample was similar to that in Figure 4a,b. With the increase in temperature, the elastic modulus of samples with high elastic modulus decreased first, then increased with the temperature increase, and started to elevate significantly after 70 °C. However, after the LP hydrolysis at 10,000 U/g concentration, it increased significantly at a higher temperature (after 75 °C). The generation of network structure in cake by denaturation of proteins during heating caused an obvious rise in storage modulus. There are many factors that affect the elastic modulus change of the system during heating, such as composition, heating rate, and salt ion effect. [4,32] Batter as a mixed system, during this experiment, is only affected by EY pollution and enzymatic hydrolysis, indicating that the density change caused by air mixing is the most prominent influence on the batter properties. Therefore, it is speculated that less air mixing is not conducive to the formation of network structure during the heating process of cake, and the inability to form mature network structures at higher temperatures may also cause gas expansion escape. The addition of EY caused the elastic modulus to rise faster in the temperature change process, which was not conducive to forming a good paste gel network. The alteration of foaming capacity induced by enzymatic hydrolysis can affect this result.

#### 3.4.2. Bubble Morphology Analysis

**Figure 5:**
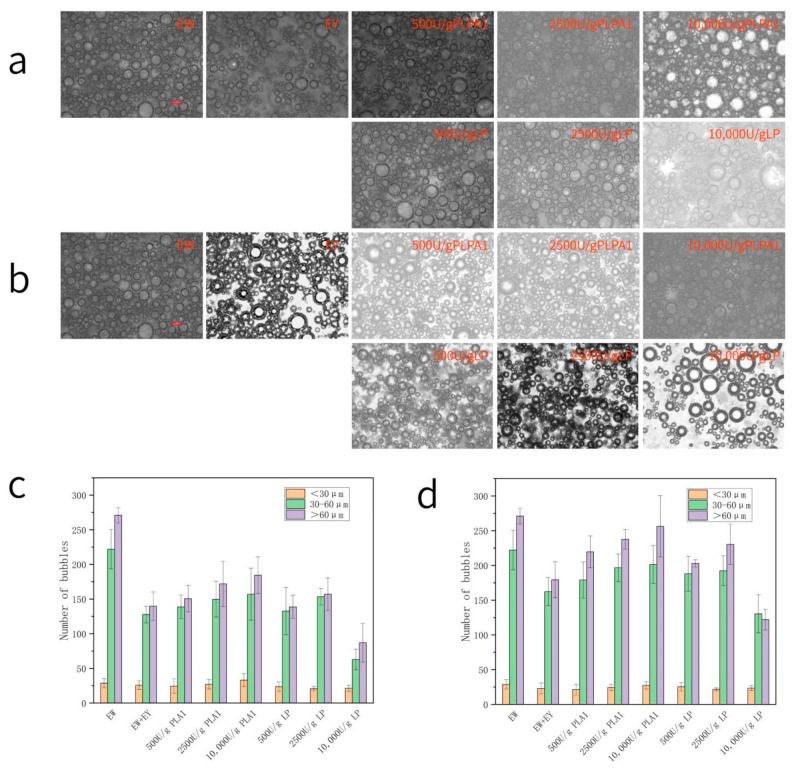
Microstructures of fresh batters. Samples prepared by 0.5 wt% yolk-contaminated EW dispersions (**a**) and 1 wt% yolk-contaminated EW dispersions. (**b**) Number of babbles in different diameter ranges in batter samples mixed with 1% (**c**) and 0.5% (**d**) egg yolk.

Bubbles form the discontinuous phase in the batter, and tiny uniform bubbles are necessary to form a cake with the desired volume and quality [33]. The amount and size distribution of incorporated air cells are essential to the volume and texture of cakes [34]. The first step in making angel food cake stick by this method was to stir the EW to form the initial foam separately. Producing as much foam as possible is critical to the paste density in this process, because no additional bubbles will form during the mixing process [35]. The number and size uniformity of foam was closely related to the foaming characteristics and foam stability in Figure 5. Foam with good foaming had more bubbles and was more uniform. In the process of mixing protein foam, sugar and flour, higher foam stability helped to avoid the bursting and disproportionation of foam after adding sugar and flour. Therefore, the influence of EY mixing and LP or PLPA1 hydrolysis at different concentrations on the bubble shape came from their influence on the EW foaming property and foam stability.

#### 3.4.3. Batter Specific Density

**Figure 6:**
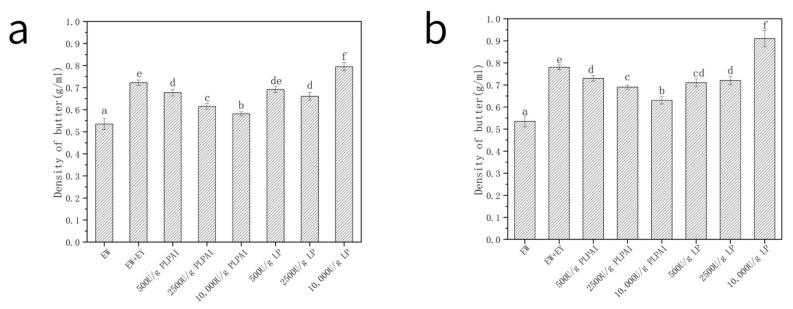
Batter densities contaminated by EY after hydrolysis. EW dispersion contaminated by 0.5 wt% (**a**) or 1 wt% (**b**) EY is hydrolyzed by PLPA1 or LP with different concentrations (500 U/g, 2500 U/g, 10,000 U/g). (Samples were prepared from and corresponded to the egg white solution in Table 2). Different letters for the same parameter indicate significant differences (*p* < 0.05) between treatments in the two figures respectively.

The density of the batter is a crucial factor in determining the volume of the cake. The mixture of EY significantly increased the density of the batter—except that the 10,000 U/g concentration of LP, PLPA1 and the density of the batter prepared from the LP-treated samples decreased. The density of the batter was mainly affected by the volume of air mixed in the batter (Figure 6). The air source in the batter depended on the foaming ability of EW protein to trap large volumes of air into the batter when whipping. According to Figure 5a,b, the photographs showed a dense amount of bubbles; a sample with a greater number of bubbles (according to Figure 5c,d) whose paste density was low, with gas mixed in, reduced batter density.

### 3.5. Characteristics of Cakes

#### 3.5.1. Cake Section and Specific Volume

Cake volume was the key factor in evaluating foam cake. The volume increases during baking as a result of steam production, and the temperature increase is low in fat-free foam-type cakes. Thus, the volume of fat-free cake systems (for example, angel food cakes) is mainly determined by gas cell incorporation during mixing and steam production during baking. Therefore, the amount of gas in the batter is the key factor in producing a larger cake.

The amount of gas added can be determined by the density of the batter [35]. Due to the release of gaseous fluid during baking, the pressure inside the gas cells of the cake batter increased. They rapidly expanded, and their extensible cell walls supported the expansion [36]. When baking is done to produce a good quality product, the outward pressure from bubble expansion cannot exceed the rate of protein coagulation and starch gelatinization [37]. Bubbles would coalesce and escape the product before a strong matrix would form if the bubble pressure were too high. Therefore, the gas retention before the formation of a stable network structure was also an important factor affecting the cake volume during the baking process; tiny and uniform bubbles were more stable during heating. When starch gelatinization and protein denaturation were delayed, the oven rise continued for a longer period, forming higher-volume cakes [38]. Figure 4c,d show that the change in storage modulus could reflect, to some extent, the change in batter structure during the heating process. The elastic modulus can rise quickly and earlier or maintain a high elastic modulus, which helps prevent thermal expansion overflow. The modulus of the sample hydrolyzed with 10,000 U/g LP and mixed with 1%wt EY increased at the higher temperature. The bubbles in the sample paste were large and uneven. Finally, the cake was almost not baked, which may be due to the expansion and dissipation of a small amount of gas before the formation of a stable structure. Except for 10,000 U/g LP hydrolysis, other concentrations of PLPA1 and LP restored the volume of cake that had been reduced by mixing EY to a certain extent, and the recovery effect increased with the increase of concentration. The effect of enzymes on foaming and foam stability can explain this.

#### 3.5.2. Textural Properties

**Figure 7:**
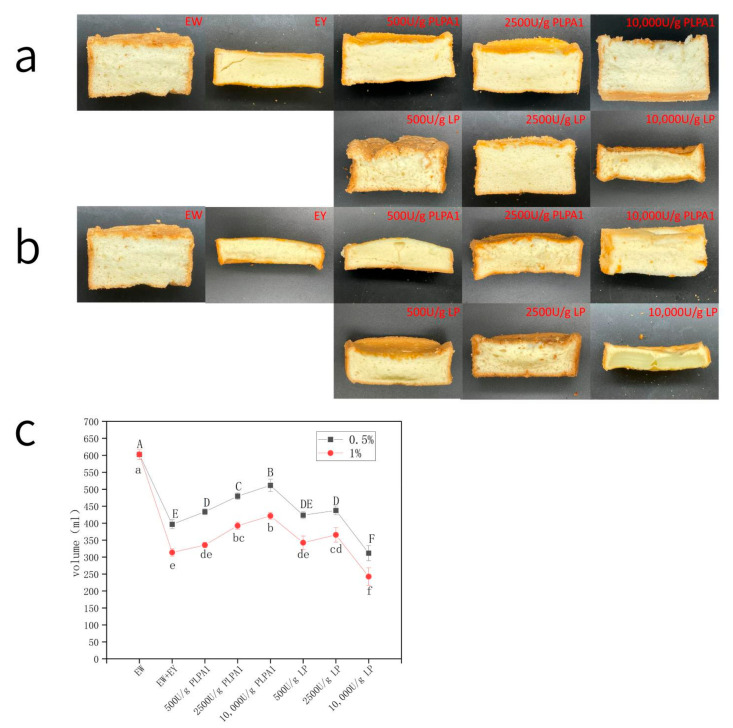
Cross section of angel cake sample. Samples mixed with (**a**) 0.5%wt EY and (**b**) 1%wt EY; (**c**) Volume of angel food cake. (black filled square represents the amount of contamination mixed with 0.5% EY; red filled circle represents the amount of contamination mixed with 1% EY). Different letters for the same parameter indicate significant differences (*p* < 0.05) between treatments.

Texture is an important factor in consumer acceptance of baked products [39]. Hardness is the peak force required to attain a particular strain during the first compression, reflecting the energy of biting a cake and the firmness of a cake [40]. As shown in Table 3, the hardness of the cake was significantly increased when EY was mixed, and the hardness of 1.0%wt mixed cake was higher than that of 0.5%wt mixed cake. The hardness of samples hydrolyzed by PLPA1 decreased significantly, the extent of which increased with a higher concentration. The hardness of the sample hydrolyzed with 500 U/g or 2500 U/g LP decreased, but the sample hydrolyzed with 10,000 U/g LP significantly increased. Greater cake hardness was attributed to lower volume and specific volume (Figure 7) [41,42]. The lower hardness meant a fluffy texture desirable for angel food cake. Compared with Figure 6 and Figure 7, the hardness conformed to this rule, indicating that the low hardness of the cake came from the fluffy network structure and the large volume of the baking paste containing more bubbles. Cohesiveness is defined as the internal resistance in the food structure [43]. When the fermented EW was utilized, the cake crumbs exhibited a lower springiness and cohesiveness (Table 3), resulting in an undesirable soggy structure and lower quality [44,45]. The viscosity of different samples had a certain convergence with the hardness. The mixture of EY increased the viscosity of the cake; PLPA1 enzymatic hydrolysis can reduce the viscosity of cake, and the degree of viscosity reduction increased with the increase of enzymatic hydrolysis concentration. The cake viscosity of samples hydrolyzed with 500 U/g or 2500 U/g LP decreased, but that of samples hydrolyzed with 10,000 U/g LP increased significantly. This may be caused by the formation of a relatively high moisture content in the cake and an immature network, leading to a moist texture of the bread. Springiness represents the ability of food to recover to its original shape after deformation [40]. The mixture of EY reduced the elasticity of the cake. When 0.5%wt EY was mixed in, the elasticity of the cake was increased by PLPA1 hydrolysis, and the elasticity was similar to that of pure EW. The elasticity was increased by LP hydrolysis at 500 U/g or 2500 U/g concentration but significantly decreased by LP hydrolysis at 10,000 U/g. After enzymolysis with 1%wt concentration sample, the elasticity of the 500 U/g or 2500 U/g PLPA1 sample did not increase, and the elasticity of the 10,000 U/g PLPA1 sample returned to the level of the blank group. The elasticity of 500 U/g or 2500 U/g LP hydrolyzed samples returned to the blank level, and the LP at 10,000 U/g concentration decreased significantly (Table 3). Low springiness would result in a soggy structure of the angel food cake [44,45], but this was only partially consistent with our previous results. The elasticity may be affected by many factors. At a certain high hardness, a dense structure could rebound the cake, and the experimental results may also be affected by certain viscosity. Cohesiveness is defined as the internal resistance in the food structure [43]. The result was similar to elasticity. Chewiness is defined as the energy required to chew food before swallowing [41]. The trend of adhesive performance was similar to that of hardness, as chewiness is determined by hardness [46]. As shown in Table 3, except for the samples mixed with 1% EY, which were hydrolyzed by 10,000 U/g PLPA1 at a concentration of 10,000 U/g that was more affected by elasticity and showed a different change trend from the sample hardness, the samples were more affected by hardness. The change rule of the chewiness of cakes affected by EY pollution and enzymatic hydrolysis was similar to the change rule of hardness. Adhesiveness was negatively related to the freshness of bakery products [47]. Research showed that low hardness, high bulkiness, and high elasticity were the high-quality standards for angel cakes [42,43]. Comprehensive texture characteristics, PLPA1 hydrolysis, and 500 U/g or 2500 U/g concentration of LP hydrolysis in cake samples mixed with 0.5% or 1% EY showed an optimization effect. A concentration of 100,000 U/g of LP could lower the quality (Table 3).

### 3.6. Correlation Analysis

Through inter-group analysis of the five groups of data in Table 4, it was found that the volume of angel food cake was positively correlated with the foaming ability of raw egg white dispersion and significantly negatively correlated with the stability of the foam. The batter’s density and the cake’s hardness were significantly negatively correlated with the foaming ability of the raw egg white dispersion and the egg foam’s stability. Large volume and low hardness are the high-quality characteristics of angel food cake. Therefore, egg white dispersion’s foaming ability and foam stability determine its product quality. Therefore, it is speculated that the appropriate concentration of phospholipase A1 and fat hydrolysis can improve the foaming ability.

## 4. Conclusions

Different concentrations (500 U/g, 2500 U/g, 10,000 U/g) of PLPA1 or LP hydrolyzed EY affected its particle size distribution and zeta potential. Foaming, PLPA1 or LP at different concentrations (500 U/g, 2500 U/g, 10,000 U/g) hydrolyzed EY at different concentrations (0.5%wt or 1.0%wt). Contaminated industrial EW-based system foaming, batters and cakes have been examined. Both PLPA1 and LP increased the absolute potential of EY. The absolute value increased significantly as the degree of enzymatic hydrolysis increased. The particle size distribution revealed that PLPA1 and LP strongly impacted particle alterations in EY. PLPA1 was primarily responsible for progressive HDL breakdown and LDL release. LP decomposed the particles and reassembled them into a single particle size, which became smaller as the degree of enzymatic hydrolysis increased. PLPA1 restored the foaming property and foam stability of EW dispersion polluted by EY. Furthermore, the recovery was more significant with the increase in enzyme concentration. LP with concentrations of 500 U/g and 2500 U/g improved the foaming characteristics and foam stability of yolk-contaminated protein dispersions, while LP at a concentration of 10,000 U/g significantly reduced the foaming characteristics and foam stability. The specific density of the batter, the volume and the hardness of the cake were related to the foaming property and foam stability. The foaming property was high. The foam stabilized dispersion had a low specific density of batter, large cake volume, uniform bubble size, and soft cake texture. The results showed that PLPA1 and appropriate concentrations of LP helped optimize the quality of angel food cake produced from EY-contaminated EW and that 10,000 U/g PLPA1 was optimal in the experiment.

## Figures and Tables

**Table 1:** Proportions of components in enzymatic EY system (The density of PLPA1 and LP solution is 1 g/mL).

Sample Type	EY (g)	LP (mL)	PLPA1 (mL)	PBS (mL)
EY	5	0	0	5
500 U/g PLPA1	5	0	0.25	4.75
2500 U/g PLPA1	5	0	1.25	3.75
10,000 U/g PLPA1	5	0	5	0
500 U/g LP	5	0.25	0	4.75
2500 U/g LP	5	1.25	0	3.75
10,000 U/g LP	5	5	0	0

**Table 2:** Composition of enzymatic yolk-contaminated EW system (The density of PLPA1 and LP. solution is 1 g/mL).

Sample Type		EW (g)	EY (g)	LP (mL)	PLPA1 (mL)	PBS (mL)
	EW	98	0	0	0	2
0.5% EY contaminated	EW + EY	98	0.5	0	0	1.5
	500 U/g PLPA1	98	0.5	0	0.025	1.475
	2500 U/g PLPA1	98	0.5	0	0.125	1.375
	10,000 U/g PLPA1	98	0.5	0	0.5	1
	500 U/g LP	98	0.5	0.025	0	1.475
	2500 U/g LP	98	0.5	0.125	0	1.375
	10,000 U/g LP	98	0.5	0.5	0	1
1.0% EY contaminated	EW + EY	98	1	0	0	1
	500 U/g PLPA1	98	1	0	0.05	1.05
	2500 U/g PLPA1	98	1	0	0.25	0.75
	10,000 U/g PLPA1	98	1	0	1	0
	500 U/g LP	98	1	0.05	0	0.95
	2500 U/g LP	98	1	0.25	0	0.75

**Table 3:** Texture profile analyses of angel food cakes obtained from yolk-contaminated EW through different enzymolysis.

Sample Type		Hardness/N	Adhesiveness/Nmm	Cohesiveness	Springiness	Chewiness/mj
0.5% EY contaminated	EW	10.72 ± 1.73 ^f^	1.78 ± 0.11 ^c^	0.83 ± 0.00461 ^a^	0.91 ± 0.0077 ^ab^	79.52 ± 12.60 ^g^
	EY	40.15 ± 4.21 ^b^	5.82 ± 0.13 ^b^	0.77 ± 0.013 ^b^	0.86 ± 0.0027 ^c^	259.27 ± 28.49 ^b^
	500 U/g LP	26.54 ± 7.47 ^cd^	2.88 ± 0.17 ^c^	0.86 ± 0.0026 ^a^	0.92 ± 0.011 ^ab^	211.15 ± 6.52 ^cd^
	2500 U/g LP	15.97 ± 1.96 ^ef^	3.11 ± 0.17 ^c^	0.84 ± 0.018 ^a^	0.89 ± 0.029 ^b^	117.85 ± 14.15 ^fg^
	10,000 U/g LP	79.51 ± 8.01 ^a^	9.86 ± 3.04 ^a^	0.75 ± 0.044 ^b^	0.80 ± 0.0025 ^d^	466.51 ± 20.42 ^a^
	500 U/g PLPA1	31.66 ± 26.37 ^c^	1.71 ± 0.011 ^c^	0.86 ± 0.0046 ^a^	0.92 ± 0.0059 ^a^	246.10 ± 23.69 ^bc^
	2500 U/g PLPA1	23.23 ± 1.98 ^de^	2.02 ± 0.198 ^c^	0.85 ± 0.0061 ^a^	0.91 ± 0.0004 ^ab^	174.61 ± 41.83 ^de^
	10,000 U/g PLPA1	19.41 ± 0.065 ^de^	1.86 ± 0.08 ^c^	0.83 ± 0.61 ^a^	0.91 ± 0.00026 ^ab^	143.65 ± 116.15 ^ef^
1.0% EY contaminated	EW	10.72 ± 1.73 ^f^	1.77 ± 0.11 ^f^	0.83 ± 0.0030 ^a^	0.91 ± 0.00042 ^a^	79.43 ± 12.72 ^e^
	EY	67.72 ± 9.41 ^b^	8.30 ± 5.93 ^b^	0.77 ± 0.0093 ^b^	0.81 ± 0.033 ^b^	416.23 ± 77.97 ^b^
	500 U/g LP	53.70 ± 8.19 ^c^	5.81 ± 0.38 ^c^	0.83 ± 0.018 ^a^	0.90 ± 0.0043 ^a^	395.28 ± 60.21 ^bc^
	2500 U/g LP	38.39 ± 5.84 ^de^	5.36 ± 0.57 ^cd^	0.84 ± 0.014 ^a^	0.91 ± 0.39 ^a^	294.99 ± 53.54 ^cd^
	10,000 U/g LP	202.91 ± 8.58 ^a^	10.46 ± 1.74 ^a^	0.73 ± 0.012 ^c^	0.74 ± 0.05 ^c^	1063.88 ± 89.32 ^a^
	500 U/g PLPA1	50.39 ± 6.43 ^cd^	4.92 ± 0.30 ^cd^	0.77 ± 0.013 ^b^	0.81 ± 0.000056 ^b^	305.58 ± 6.95 ^bc^
	2500 U/g PLPA1	38.39 ± 1.25 ^de^	4.29 ± 0.54 ^d^	0.83 ± 0.0081 ^a^	0.80 ± 0.0041 ^b^	231.31 ± 0.46 ^d^
	10,000 U/g PLPA1	35.24 ± 4.06 ^e^	2.19 ± 0.48 ^e^	0.83 ± 0.0048 ^a^	0.91 ± 0.00050 ^a^	261.46 ± 30.80 ^d^

The superscript letters (^a, ^^b, ^^c, ^^d, ^^e, ^^f, ^^g^) represents that means within columns with similar letters are not significantly different (*p* > 0.05).

**Table 4:** Results of correlation analysis.

	Foam Capacity (mL/g)	Drainage Rate (%)	Batter Specific Density (g/mL)	Angel Cake‘s Volume (mL)	Angel Cake‘s Hardness (N)
Foam capacity (mL/g)	1				
Drainage rate(%)	−0.841 **	1			
Batter specific density(g/mL)	−0.925 **	0.821 **	1		
Angel cake‘s volume(mL)	0.87 **	−0.874 **	−0.85 **	1	
Angel cake‘s hardness(N)	−0.562 **	0.636 **	0.72 **	−0.72 **	1

Correlations among different indicators were carried out using Pearson’s correlation coefficient in bivariate linear correlations. ** *p* < 0.01.

## Data Availability

The data presented in this study are available on request from the corresponding author.
